# A Rare Case of Ruptured Sinus of Valsalva Aneurysm With Noncoronary Sinus to the Right Ventricle

**DOI:** 10.1016/j.atssr.2024.04.019

**Published:** 2024-05-09

**Authors:** Joo Kunihiko, Ochiai Yoshie, Motomatsu Yuma, Tokunaga Shigehiko

**Affiliations:** 1 Department of Cardiovascular Surgery, JCHO Kyushu Hospital, Kitakyushu City, Japan

A 34-year-old man with a perimembranous outlet ventricular septal defect and aortic regurgitation had acute heart failure after congenital intestinal atresia treatments. Echocardiography revealed a new continuous left-to-right shunt in the right ventricle (Video 1). A ruptured sinus of Valsalva aneurysm (SVA; arrows in Figure) was suspected. Computed tomography and 3-dimensional computer graphic software (Viewtify; SCIEMENT, Inc) analysis confirmed that the noncoronary sinus (NCS) ruptured into the right ventricle (Figures A, B; Video 2).

**Figure 1:**
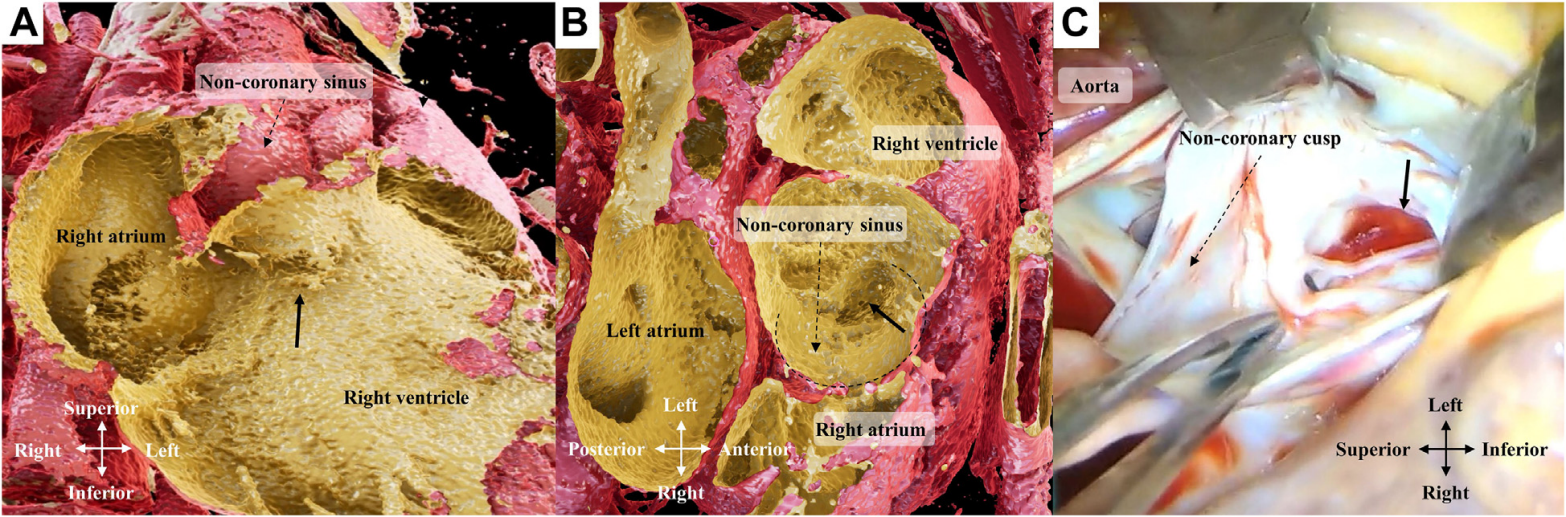

Intraoperative findings indicated NCS rupture (Figure C; Video 3), repaired with a 5-0 monofilament double-running suture parallel to the aortic annulus through aortotomy; the ventricular septal defect was closed with a knitted Dacron patch. Aortic valve replacement with a bioprosthetic valve was performed for residual aortic regurgitation caused by noncoronary cusp degeneration.

Sakakibara and Konno^1^ proposed the first formal SVA classification; however, NCS–to–right ventricle communication was not observed. Moreover, 45 of 257 patients with SVA had lesions at the NCS, and only 3 ruptured into the right ventricle.^2^ Three-dimensional imaging with Viewtify aided in understanding this rare anatomic structure.
